# Specific expression profile of follicular fluid-derived exosomal microRNAs in patients with diminished ovarian reserve

**DOI:** 10.1186/s12920-023-01756-9

**Published:** 2023-11-30

**Authors:** Kai-Yuan Shen, Xiao-Li Dai, Shun Li, Fen Huang, Li-Qun Chen, Ping Luo, Xiao-Li Qu

**Affiliations:** 1 Department of Reproductive Medicine, Liuzhou People’s Hospital, Liuzhou, Guangxi, 545006 People’s Republic of China; 2 Research service office, Liuzhou People’s Hospital, Liuzhou, Guangxi, 545006 People’s Republic of China; 3 Liuzhou Key Laboratory of Reproductive and Genetic Metabolic Diseases, Liuzhou, Guangxi 545006 People’s Republic of China

**Keywords:** Diminished ovarian reserve, Exosomes, MicroRNA, Follicular fluid, Next-generation sequencing

## Abstract

**Background:**

Diminished ovarian reserve (DOR) is defined as a reduction in ovarian reserve and oocyte quality. The pathophysiology of DOR has not been completely explained as of yet. Scholars have uncovered a large number of exosomes that have been detected in follicular fluid, and exosomal miRNAs have been proven to play a critical role in controlling ovarian disorders and follicle formation. We focused on the expression profile of follicular fluid-derived exosomal microRNAs (miRNAs) and attempted to understand if their role is connected to the pathomechanism of DOR.

**Methods:**

The follicular fluid-derived differentially expressed exosomal miRNAs (DEmiRs) between patients with DOR and those with normal ovarian function were investigated using the next-generation sequencing (NGS) method. The main metabolic and signaling pathways of DEmiRs were identified using the KEGG pathway database, disease ontology (DO) analysis, and gene ontology (GO) analysis. In the end, a Protein-Protein Interaction (PPI) network was built to search for exosomal miRNAs and their target genes that were potentially strongly connected with DOR.

**Results:**

In comparison to normal controls, 52 DEmiRs were discovered in follicular fluid-derived exosomes of DOR patients, of which 19 were up-regulated and 33 were down-regulated (|log2(fold change) |>2, *P* < 0.05). GO, DO analysis, and the KEGG pathway database revealed that many of these DEmiRs have broad biological roles that are connected to ovarian function and disorders. The top ten DEmiRs in terms of expression were then chosen for miRNA-mRNA interaction analysis. Totally, 8 experimentally supported miRNAs (hsa-miR-1246, hsa-miR-483-3p, hsa-miR-122-5p, hsa-miR-130b-3p, hsa-miR-342-3p, hsa-miR-625-3p, hsa-miR-675-3p, and hsa-miR-134-5p) and 126 target genes were filtrated by utilizing Cytoscape software. The module analysis findings of the PPI network showed that the main module cluster with a score > 6.0 (MCODE score = 15) had six hub genes, including IGFR, VEGFA, KRAS, ERBB2, RHOA, and PTEN (MCODE score = 11.472).

**Conclusion:**

Our data suggested a special expression profile of follicular fluid-derived exosomal miRNAs in patients with DOR, which was probably correlated to ovarian dysfunction and follicle formation. These results may give a unique insight into a better understanding of the molecular process in the pathogenesis of DOR or other ovarian diseases.

**Supplementary Information:**

The online version contains supplementary material available at 10.1186/s12920-023-01756-9.

## Introduction

Diminished ovarian reserve (DOR) is characterized by a decline in ovarian reserve and oocyte quality and is one of the key concerns linked with failure in assisted reproductive technology (ART). The pathogenic mechanism of DOR is highly complicated and is most likely multiple, and recognized causes include genetic, iatrogenic, environmental, autoimmune, and aging [1, 2]. In recent years, more and more novel pathogenic mechanisms in DOR have been revealed, including oxidative stress injury, mitochondrial dysfunction, energy metabolism damage, autophagy, and endoplasmic reticulum stress [3, 4]. Although efforts have been made in DOR, the pathological and molecular mechanisms are still poorly understood.

Exosomes, which make up a large portion of extracellular vesicles (EVs), are produced by practically all cell types and range in size from 30 to 150 nm in diameter [5]. Particularly, the genetic material and functional proteins found in cell-derived exosome cargo, such as long non-coding RNAs (lncRNAs) and microRNAs (miRNAs), can be used locally or transmitted to target cells in a stable manner. Exosomes can thus play a role in the modulation of a number of physiological and pathological processes, including immunological response, antigen presentation, cell-cell communication, and RNA transport [6, 7]. miRNA has a length of 18–25 nucleotides [8] and is known to play a significant role in the majority of biological processes, including tumorigenesis, cell proliferation, cell differentiation, and cell death [9]. Exosomal miRNAs are considered potential diagnostic biomarkers for such as conditions cancer [10], Acute Myocardial Infarction [11] and Type II Diabetic Nephropathy [12]. Besides, other researchers have considered the combination of electrochemical biosensors in exosome analysis to better evaluate the occurrence of diseases in early-stage [13]. Various pieces of evidence point to a tight connection between exosomal miRNAs and female reproductive disorders such as ovarian cancer [14, 15], polycystic ovary syndrome (PCOS) [16], and endometriosis [17]. The proper follicular development and oocyte maturation depend on the follicular fluid (FF), which creates an essential microenvironment. Exosomal miRNAs were found in the follicular fluid of human, bovine and other species, according to previous studies [18–20], and they may closely relate to the development of ovarian follicles. But the contribution of follicular fluid exosomal miRNAs to female infertility hasn’t been fully clarified.

Here, we used next-generation sequencing to explore the exosomal miRNAs that were differentially expressed in the follicular fluid of DOR patients. We concentrate on FF exosomal miRNA expression profile in DOR and look for any particular exosomal miRNAs that may be significantly influencing the pathological development of DOR. The results may offer fresh insights into possible biomarkers and signaling pathways to treat female infertility brought on by ovarian malfunction, as well as assistance in our understanding of the molecular mechanism of exosomal miRNAs in DOR.

## Materials and methods

### Patients

The patients were divided into two groups according to the ovarian evaluation (Normal and DOR groups). Human follicular fluid (FF) samples were collected from women undergoing infertility therapy with in vitro fertilization-intracytoplasmic sperm injection (IVF-ICSI) at the Human Assisted Reproduction Medical Center of Liuzhou People’s Hospital. The inclusion criteria for this study were: (1) Patients who are younger than 35 years, ovulated, have normal basal hormone levels, and antral follicles count (AFC) at the range of 7 ~ 20 on the 2–3 days of menstruation due to male factor infertility or tubal factors were selected into Normal group. (2) DOR was diagnosed based on the well-accepted international diagnostic criteria [21]. Patients are younger than 35 years, ovulated, and have more than two points as follows: (i) AFC ≤ 5–7 on the 2–3 days of menstruation; (ii) anti-mulller tube hormone (AMH) < 0.5-1.1ng/ml; and (iii) basal FSH > 10 IU/L were selected into DOR group. The exclusion criteria for this study were: factors that could affect oocyte quality include endometriosis, polycystic ovaries, hyperprolactinemia, pelvic oviduct inflammation, and ovarian insufficiency, with habits of smoking and drinking, or females with dyslipidemia and diabetes mellitus. Clinical characteristics are summarized in Table 1.

**Table 1:** Clinical characteristics of DOR and Normal patients. This table divides into five columns and eleven rows, first row contains Parameters, DOR (n = 15), Normal (n = 15), t, and *P*, parameters contain Age, BMI (kg/m2), Basal serum FSH (IU/L), Basal serum LH (IU/L), Basal serum PRL (ng/mL), Basal serum E2 (pmol/L), Basal serum T (nmol/L), Basal serum P (nmol/L), AMH (ng/mL), AFC. At the bottom of the table are the full names of parameter abbreviations

Parameters	DOR (n = 15)	Normal (n = 15)	t	*P*
Age	34.73 ± 2.99	33.47 ± 2.53	-1.253	0.221
BMI (kg/m^2^)	22.38 ± 3.09	20.30 ± 2.19	-2.126	0.042
Basal serum FSH (IU/L)	9.04 ± 2.83	6.52 ± 1.02	-3.245	0.005
Basal serum LH (IU/L)	4.63 ± 2.49	5.08 ± 1.55	0.593	0.558
Basal serum PRL (ng/mL)	324.42 ± 127.81	415.99 ± 111.59	2.09	0.046
Basal serum E2 (pmol/L)	255.69 ± 236.9	170.88 ± 66.05	-1.336	0.2
Basal serum T (nmol/L)	0.78 ± 0.47	0.86 ± 0.51	0.484	0.632
Basal serum P (nmol/L)	1.64 ± 1.57	1.28 ± 1.56	-0.628	0.535
AMH (ng/mL)	0.58 ± 0.30	3.18 ± 1.25	7.851	< 0.001
AFC	5.40 ± 1.45	15.53 ± 4.84	7.768	< 0.001

Note: All results are presented as the mean ± SD

Abbreviations: BMI, body mass index; FSH, follicle-stimulating hormone; LH, luteinizing hormone; PRL, prolactin; E2, estradiol; T, testosterone; P, progesterone; AMH, Anti-mullerian hormone; AFC, antral follicle count

### Human FF sample preparation

The ovarian response to stimulation was monitored by measuring the serum E2 concentration and by ultrasound assessment of follicular growth. When serum E2 levels were observed to be in the range of 550.5–734.0 pmol/L and the leading follicle reached a diameter of > 18 mm, HCG (10,000 IU) was then given to induce ovulation. Oocytes were harvested after hCG injection for 34 ~ 36 h through a vaginal ultrasound-guided follicle puncture. FF collection provided the approximate volumes of follicular fluid: 10–15 ml, transferred in a clean 15-ml centrifuge tube. To eliminate follicular cell residues and any blood traces, FF was centrifuged at 3,000 × g for 10 min at room temperature. The supernatant was then immediately transferred to a clean tube and stored at -20 °C for subsequent analysis. To generate a homogenous pool of samples, for every five patients’ FF was mixed as one pooled sample.

### Exosome isolation and characterization

Exosome isolation using the Umibio® Exosome isolation kit was made on mixed FF samples in each group (Umibio, China). The reagents were added in the appropriate quantities following the manufacturer’s instructions. To obtain exosome precipitate, mixtures were vortexed and incubated for up to two hours at 4 °C and then centrifuged at 10,000×g for 60 min at 4 °C. To obtain the exosome pellets, the precipitate was purified with an Exosome Purification Filter at 3,000 g for 10 min at 4 °C. Every 200 µl of exosome pellets was diluted up to 20 ml with 1×PBS and then kept at -80 °C immediately for further analysis. The exosome identification was finished by Umibio Company, and the exosome morphology observation by using transmission electron microscopy (TEM). Exosome pellets were resuspended in 50–100 µl 2% paraformaldehyde (PFA), followed by the addition of 5 µl of the exosome solution to the formvar-Carbon copper mesh. The copper mesh was exposed to 50 µl 1% glutaraldehyde droplets for 5 min, and then thoroughly washed in 100 µl ddH2O for 2 min (Repeat 8 times). After undergoing a negative staining treatment, the mesh was placed on filter paper to dry before being examined with a TEM operating at 80 kV. The size and concentration of exosomes were determined by nanoparticle tracking analysis (NTA) using a ZetaView PMX 110 (Particle Metrix) and the accompanying software ZetaView 8.04.02. Isolated exosome samples were appropriately diluted using 1×PBS buffer and measured by NTA, which was performed on 11 positions. To calibrate the ZetaView system, 110 nm polystyrene particles were used, which were kept at a constant 27.58 °C. Exosome markers were identified using Western blot. Briefly, exosome pellets were dissolved in the protein lysis buffer, and protein concentration was determined using a Pierce™ BCA Protein Assay Kit (ThermoFisher, USA). Before being transferred to a PVDF membrane, the proteins were separated on a 15% SDS-PAGE gel. PVDF membrane was cut to a suitable size at protein molecular weight distribution (TSG101 approx. 30 ~ 50 kDa, CD63 approx. 30 ~ 60 kDa) aprior to incubation with TSG101 (ab83, abcam) and CD63 (ab193349, abcam) primary antibodies overnight at 4 °C, followed by incubation with the corresponding secondary antibodies for 1 h at room temperature. Exosome protein bands were visualized using the SuperSignal chemiluminescence system (ECL, Pierce, USA).

### Next-generation sequencing (NGS)

TRIzol® Reagent (Invitrogen, USA) was used to extract the total RNA from the exosomes under the manufacturer’s instructions. Agilent 2100 (Agilent, USA) was used to evaluate RNA concentrations and quality (RNA ≥ 1ng/µl could be utilized for further analysis). Umibio (Shanghai) Co. Ltd. and Majorbio (Shanghai) Co. Ltd. handled NGS, data production, and normalization. Briefly, for each sample, the TruseqTM Small RNA Sample Prep Kit (Illumina, San Diego, CA) was used to create the miRNA library using around 10 ng of RNA. Then the Illumina HiSeq4000 system (Illumina, San Diego, CA) was used for sequencing using 50-bp single-end reads after pooling equal amounts of small RNA libraries for gel purification. First, to get clean reads, low-quality reads, adaptor sequences, and reads with more than 10% unknown bases (N bases) were eliminated. Second, to identify known and novel miRNA, siRNA, piRNA, and sRNA, all filtered small RNA was subjected to adaptor sequence and read length distribution analysis, and aligned against the Rfam (12.1, http://Rfam.sanger.ac.uk/) and miRbase (miRbase 21, http://www.mirbase.org/) databases. Finally, the expression level of miRNAs from each library was calculated by software Bowtie, RNAfold, and miRDeep2 and normalized each miRNA reads using Transcripts Per Million (TPM, TPM = miRNA reads counts *1000000/library size). DESeq2 was used to identify miRNAs that were differentially expressed (Log_2_fold change (FC) > 2 or < -2, *P*-value < 0.05 based on Benjamini and Hochberg multiple testing correction).

### Real-time quantitative PCR

For DEmiRs validation, real-time quantitative PCR (qRT-PCR) was carried out using a LightCycler 480 II (Roche, USA) using the SYBR Premix Ex TaqII (Tli RNaseH Plus) (Takara, Japan). Briefly, the total RNA from each sample was performed with the PrimeScript™ RT reagent Kit with gDNA Eraser (Perfect Real Time) (Takara, Japan) for reverse transcription. miRNA primer information is shown in Additional file 5. Each reaction was conducted in a 20-µl system with 2.0 µl of cDNA, 0.8 µl forward and reserve primer, 10 µl Power SYBR Green PCR Master Mix, 0.4 µl ROX Reference Dye II and 6.8 µl RNase-free water. The PCR conditions consisted of denaturation at 95 °C for 30 s, followed by 40 cycles of denaturation at 95 °C for 5 s and extension at 60 °C for 34 s. Human U6 small nucleolar RNA (U6-snRNA) was used as a reference gene. With three parallels in each biological replication, experiments were carried out in triplicate. The comparative threshold cycle (2^−∆∆Ct^) approach was used for the analysis.

### Target gene prediction and enrichment information

Prediction of miRNA target genes relied on the databases miRanda (http://www.microrna.org/microrna/home.do), TargetScan (http://www.targetscan.org/), and miRbase (https://mirbase.org/). The miRNA-mRNA network was constructed using Cytoscape (version 3.80) package CytargetLinker. Gene Ontology (GO), Kyoto Encyclopedia of Genes and Genomes (KEGG), and Disease Ontology (DO) analysis using databases MiEAA 2.0 (https://ccb-compute2.cs.uni-saarland.de/mieaa2/). The cut-off values were set at Clustered Genes/Total Genes ≥ 4% and *P* < 0.05, respectively.

### Protein-protein interaction (PPI) network construction and network module analysis

PPI network construction and network module analysis to explore the functional relationships of target genes. The PPI network was predicted using the Search Tool for the Retrieval of Interacting Genes (STRING; http://string-db.org) (version 10.0) online database. The PPI data was imported into Cytoscape 3.80 to construct a PPI network and perform topological analysis. The most important module and hub genes in the PPI network were identified using the molecular complex detection (MCODE) method.

### Statistical analysis

Statistical analysis was conducted using R software version 4.21. The comparison of patient characteristics in two groups was done using an unpaired t-test, with significance accepted at *P* < 0.5. All pictures in this research were visualized using R software 4.21, Cytoscape 3.80, and the Hiplot online drawing tool (https://hiplot.com.cn/).

## Results

### Clinical characteristics of DOR and normal patients

This research employed the normal patient group as a control group. The characteristics of DOR and normal patients are presented in Table 1. There was no difference concerning Age, Basal serum LH, Basal serum E2, Basal serum T, and Basal serum P level. BMI and Basal serum PRL level were statistically different between the two groups (*P* < 0.05), but FSH, AMH, and AFC levels were dramatically and significantly different between the two groups (*P <* 0.01).

### Identification of follicular fluid exosome

**Fig. 1:**
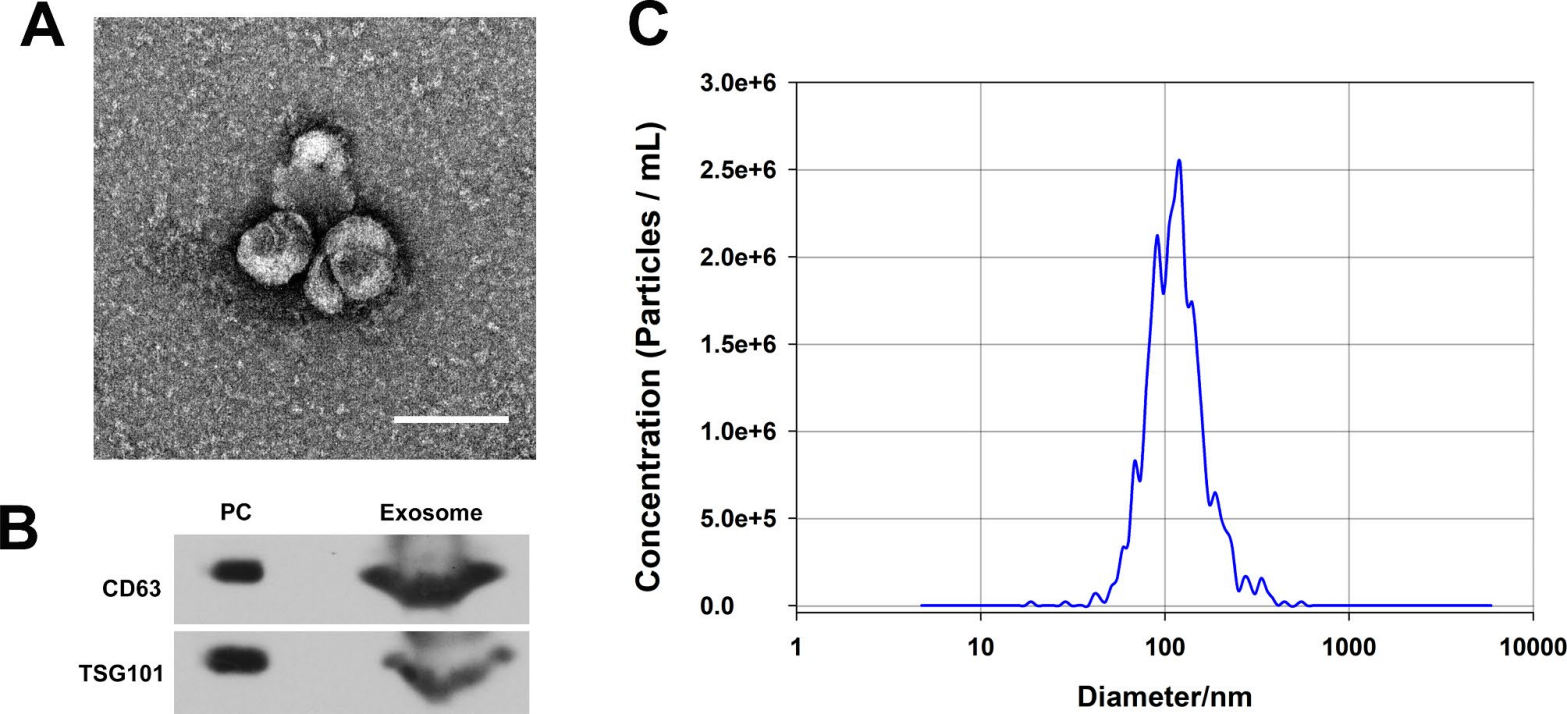
Characterization of follicular fluid-derived exosomes. (**A**) transmission electron microscopy (bar = 200 nm), (**B**) western blotting (PC: positive control; The results of CD63 and TSG101 blotting derived from different gels) and (**C**) Nanoparticle tracking analysis

Identification of the exosomes revealed that the morphology of the particles was similar to the exosome by using TEM (Fig. 1A). Using western blotting analysis, TGS101 and CD63, both of which are specific markers of exosomes, were detected in the particles (Fig. 1B). Furthermore, size distribution analysis using ZetaView PMX 110 was performed on particles to demonstrate their homogeneous size. These particles all had particle diameter distribution peaks between 80 and 150 nm (Fig. 1C).

### Global exosome miRNA profiling from NGS analysis

**Fig. 2:**
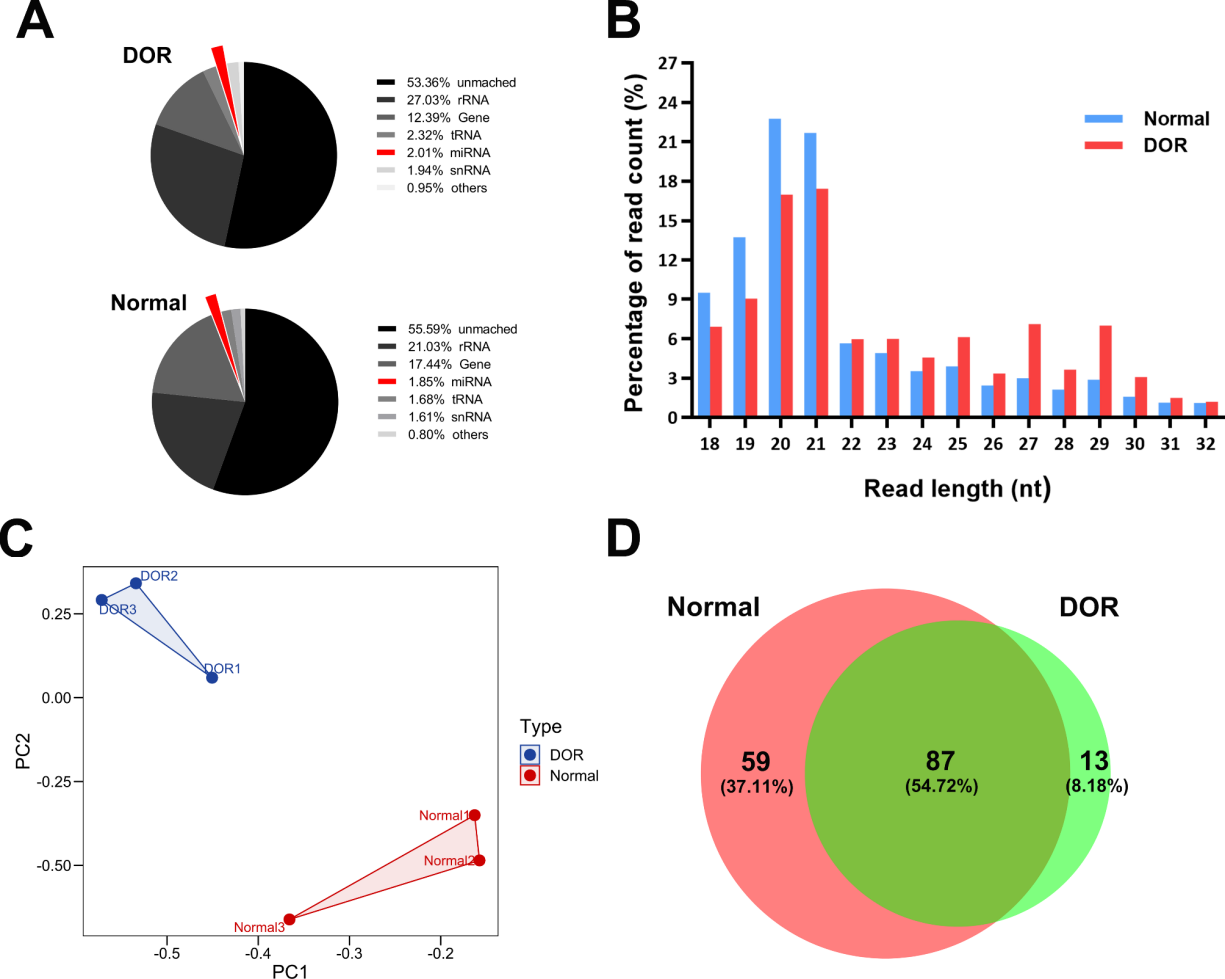
Profiling of small RNAs in DOR and Normal exosomes. **(A)** The relative abundance of different classes of small RNAs. **(B)** Size and frequency distribution of detected small RNAs (18–32 nt). **(C)** Principle component analysis of total known miRNAs in DOR and Normal exosomes. **(D)** Venn diagram showing the commonly expressed miRNAs in DOR and Normal exosomes

**Fig. 3:**
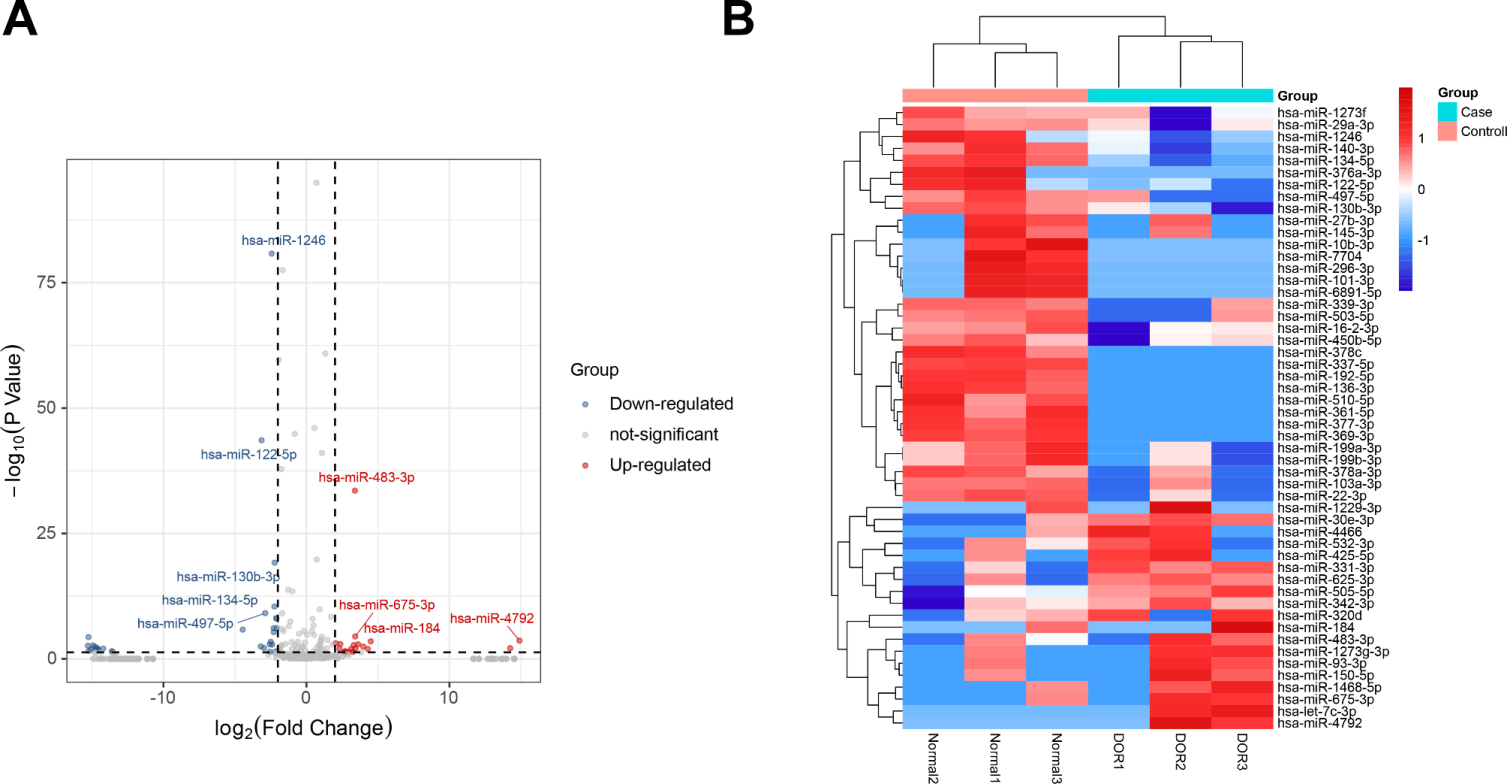
**(A)** Identifying the differentially expressed miRNAs by a volcano plot. The two vertical lines are the 2-fold change boundaries and the horizontal line is the statistical significance boundary (Adj *P*-value < 0.05). The statistically significant up-regulated and the down-regulated miRNAs with a 2-fold change ≥ 2 are marked with red and blue dots respectively. **(B)** Heatmap generated by clustering differentially expressed miRNAs in DOR and Normal exosomes. The color bar represents a relative expression, “red” indicates a high relative expression and “blue” indicates a low relative expression

Candidate exosomal miRNAs were found in the follicular fluid samples of DOR and Normal individuals using miRNA deep sequencing. After applying quality and length filters, clear readings totaling 11,508,887 were retrieved from DOR samples and 7,832,378 from Normal samples for further investigation (data not shown). After filtration, clean reads were merged into unique reads, and the species and quantity of non-miRNA sequences were determined using the Rfam database (12.1, http://Rfam.sanger.ac.uk/) and Blastn software (http://blast.ncbi.nlm.nih.gov/). The proportion of annotated miRNAs was separated as 2.01% and 1.89% in these exosomes, respectively (Fig. 2A). The length distribution of reads showed the anticipated peak at 18 to 21 nt, which corresponds to the typical miRNA length (Fig. 2B). The expression level of the known and novel miRNA in each sample was quantified, and the expression level was standardized using TPM. The principle component analysis revealed that the miRNA profiles of DOR and Normal exosomes were distinct (Fig. 2C). There were 87 common-expressed miRNAs between the two groups (Fig. 2D). Totally, 52 differentially expressed miRNAs (DEmiRs) were identified. In detail, 19 miRNAs were up-regulated, whereas 33 were down-regulated (|log_2_(foldchange)|>2, *P* < 0.05) (Fig. 3A). The hierarchical clustering analysis determined that the FF exosomes in two groups have distinct miRNA profiles (Fig. 3B). (The details of the raw data are in Additional file 1: Table S1)

### GO terms, DO terms and KEGG pathway annotation of DEmiRs

**Fig. 4:**
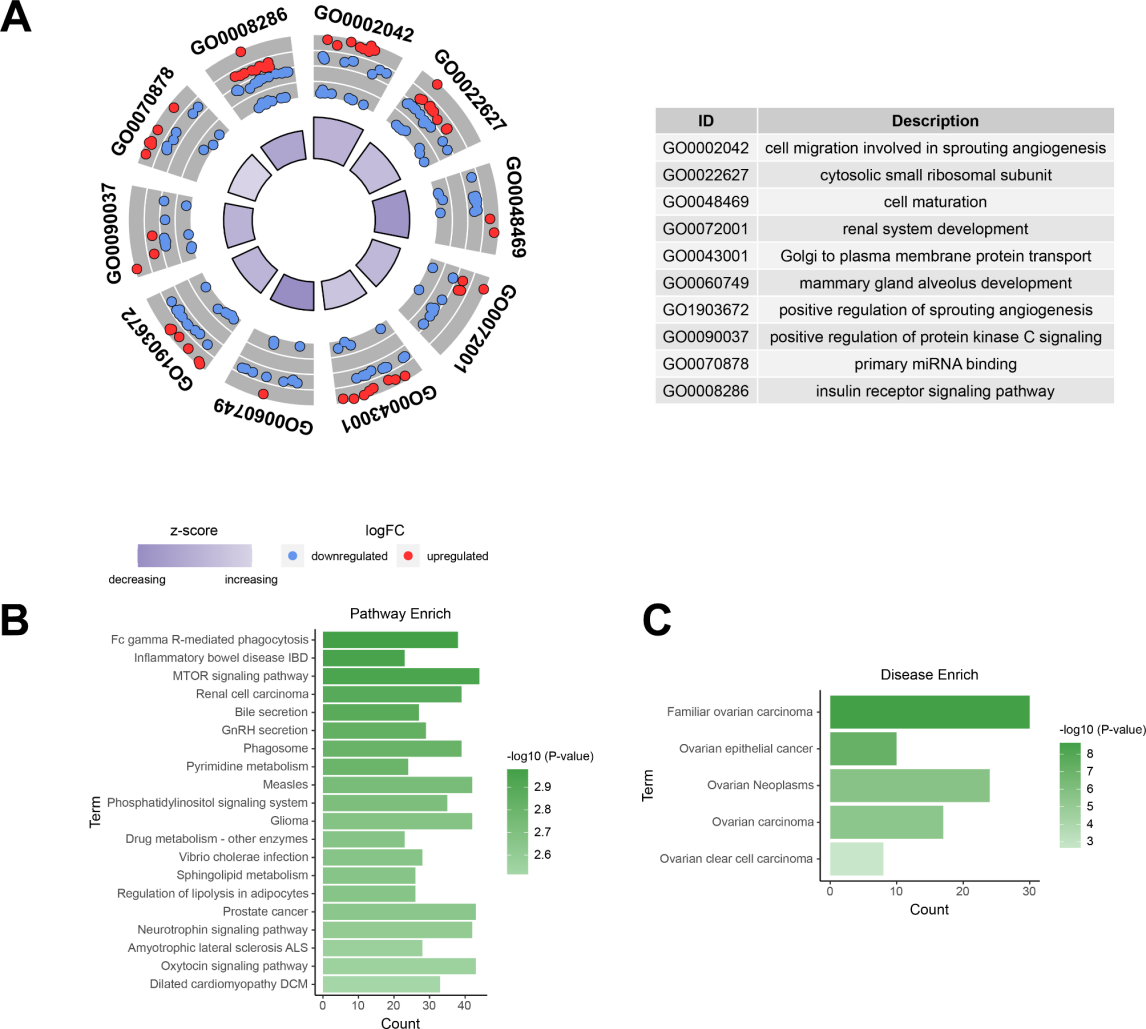
GO category, pathway analysis and DO category for DEmiRs. **(A)** The bar plot is shown in the inner ring, with the color corresponding to the z-score and the height of the bar representing the significance of the biological process (BP) term. **(B)** Results of KEGG pathway analysis for the DEmiRs. **(C)** Results of ovarian-related disease enrichment analysis for the DEmiRs.

The biological processes mediated by DEmiRs were enriched by GO annotation, with the top 10 categories being as follows: *cell migration involved in sprouting angiogenesis* (GO0002042), *cytosolic small ribosomal subunit* (GO0022627), *cell maturation* (GO0048469), *renal system development* (GO0072001), *Golgi to plasma membrane protein transport* (GO0043001), *mammary gland alveolus development* (GO0060749), *positive regulation of sprouting angiogenesis* (GO1903672), *positive regulation of protein kinase C signaling* (GO0090037), *primary miRNA binding* (GO0070878,)*insulin receptor signaling pathway* (GO0008286)(Fig. 4A). Results of the top 20 KEGG annotations of DEmiRs are shown in Fig. 4B, such as *Fc gamma R-mediated phagocytosis*, *Inflammatory bowel disease IBD*, *mTOR signaling pathway*, *Renal cell carcinoma*, *Bile secretion*, etc. According to disease ontology analysis, DEmiRs might mediate five ovarian-related diseases, including *Ovarian clear cell carcinoma*, *Ovarian Neoplasms familiar*, *Ovarian carcinoma*, and *Ovarian epithelial cancer* (Fig. 4C). (Full results of annotation in Additional file 1: Table S2-S4)

### Target genes predication and miRNA-mRNA network construction

**Fig. 5:**
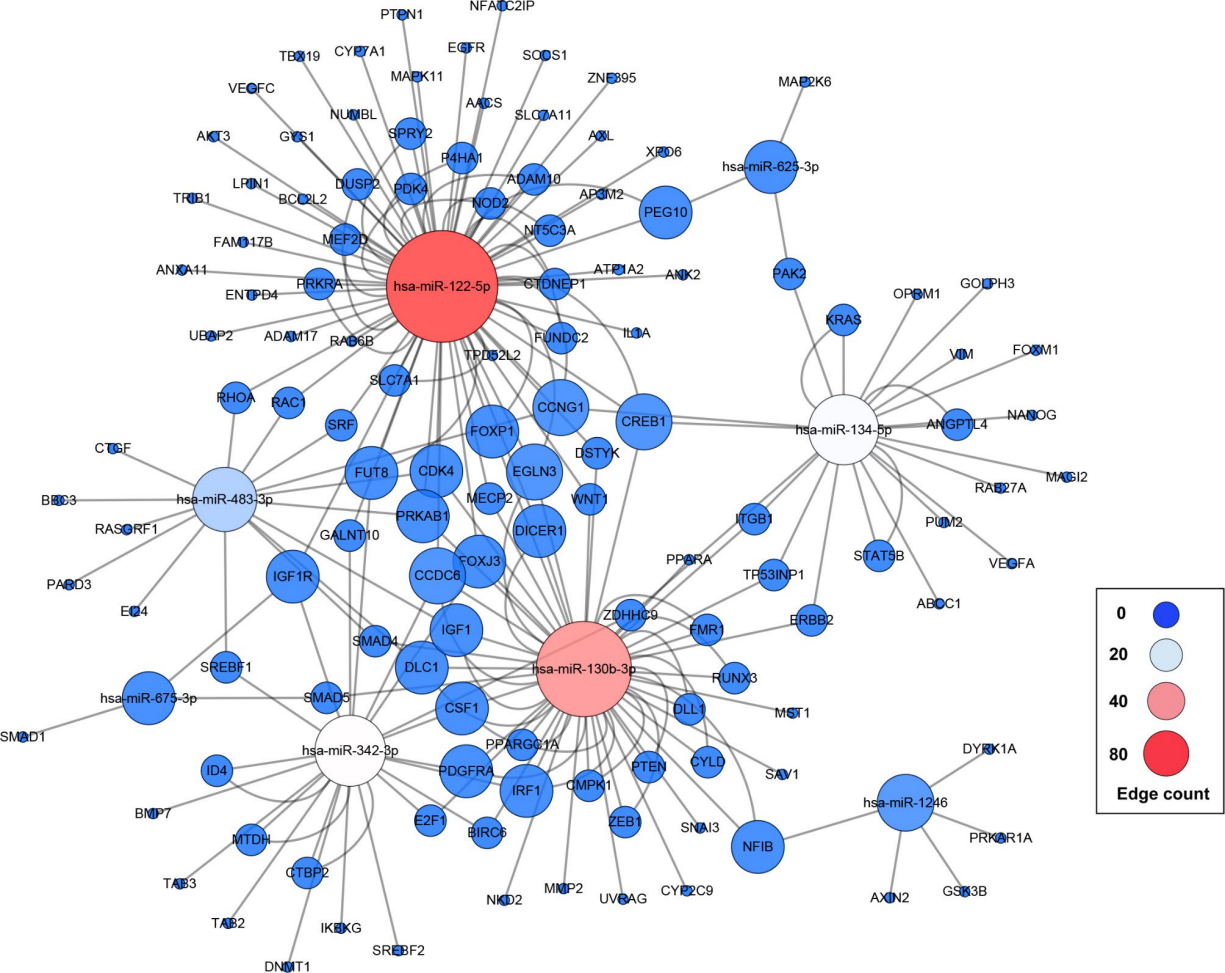
The miRNA-mRNA interaction network. The edge indicates the interaction between miRNA and predicated target mRNA. The “Edge count” is used to describe the importance of miRNA nodes in the network, the size of the circle presents a low to high Edge count

To predict target genes, the top 10 DEmiR expression levels in DOR samples were selected. Only experiment-supported DEmiRs and their target genes were chosen for further analysis using the Cytoscape add-in CyTargetLinker to build a high-credibility miRNA-mRNA network based on the miRanda, TargetScan, and miRbase databases. Totally, 8 DEmiRs (hsa-miR-1246, hsa-miR-483-3p, hsa-miR-122-5p, hsa-miR-130b-3p, hsa-miR-342-3p, hsa-miR-625-3p, hsa-miR-675-3p, hsa-miR-134-5p) and 126 target genes were filtrated (Table 2). Down-regulated hsa-miR-122-5p (Edge count = 61) and up-regulated hsa-miR-342-3p (Edge count = 21) had the greatest Edge count value of all, respectively (Fig. 5).

**Table 2:** Putative target genes of the differentially expressed miRNAs in follicle fluid-derived exosomes in patients with DOR compared to normal control subjects. This table divides into three columns and nine rows, includes four items miRNA name, miRNA expression, and Genes, every row shows the differentially expressed miRNAs and their corresponding expression and predicted target genes

miRNA name	miRNA expression	Genes
hsa-miR-483-3p	Up	RHOA, RAC1, IGF1, CDK4, BBC3, RASGRF1, SRF, CCNG1, SREBF1, PARD3, SMAD4, PRKAB1, EI24, CTGF, DLC1
hsa-miR-342-3p	Up	IKBKG, TAB2, TAB3, PDGFRA, SREBF2, CSF1, CCDC6, FOXJ3, BMP7, IGF1R, IRF1, GALNT10, MTDH, SREBF1, FUT8, CTBP2, ZDHHC9, DNMT1, ID4, BIRC6, E2F1
hsa-miR-625-3p	Up	PEG10, PAK2, MAP2K6
hsa-miR-675-3p	Up	SMAD1, SMAD5, IGF1R
hsa-miR-1246	Down	NFIB, GSK3B, AXIN2, DYRK1A, PRKAR1A
hsa-miR-122-5p	Down	MEF2D, ZNF395, CDK4, SOCS1, NT5C3A, P4HA1, PTPN1, CCNG1, DICER1, SPRY2, BCL2L2, FAM117B, DSTYK, PRKAB1, WNT1, IL1A, CTDNEP1, CREB1, LPIN1, PEG10, CYP7A1, PRKRA, FOXJ3, CCDC6, SLC7A11, TRIB1, EGLN3, NUMBL, ADAM17, AP3M2, SLC7A, FUT8, XPO6, UBAP2, TBX19, AACS, DUSP2, AXL, NOD2, ATP1A2, MAPK11, FUNDC2, AKT3, TPD52L2, ENTPD4, ANXA11, RHOA, RAB6B, FOXP1, RAC1, MECP2, GYS1, ANK2, NFATC2IP, SRF, IGF1R, GALNT10, PDK4, ADAM10, EGFR, VEGFC
hsa-miR-130b-3p	Down	UVRAG, PDGFRA, FMR1, CREB1, CSF1, CDK4, FOXJ3, MMP2, CCDC6, ZEB1, IRF1, EGLN3, TP53INP1, NKD2, CMPK1, DLL1, DICER1, PPARGC1A, SMAD4, CYLD, CYP2C9, BIRC6, E2F1, FOXP1, SMAD5, MECP2, IGF1, SNAI3, PPARA, ERBB2, SAV1, MST1, DSTYK, PRKAB1, RUNX3, WNT1, NFIB, ITGB1, DLC1, PTEN
hsa-miR-134-5p	Down	MAGI2, GOLPH3, CREB1, PUM2, NANOG, KRAS, FOXM1, TP53INP1, CCNG1, OPRM1, ANGPTL4, ZDHHC9, ABCC1, VEGFA, ERBB2, STAT5B, VIM, PAK2, ITGB1, RAB27A

### Differentially expressed miRNAs validation

**Fig. 6:**
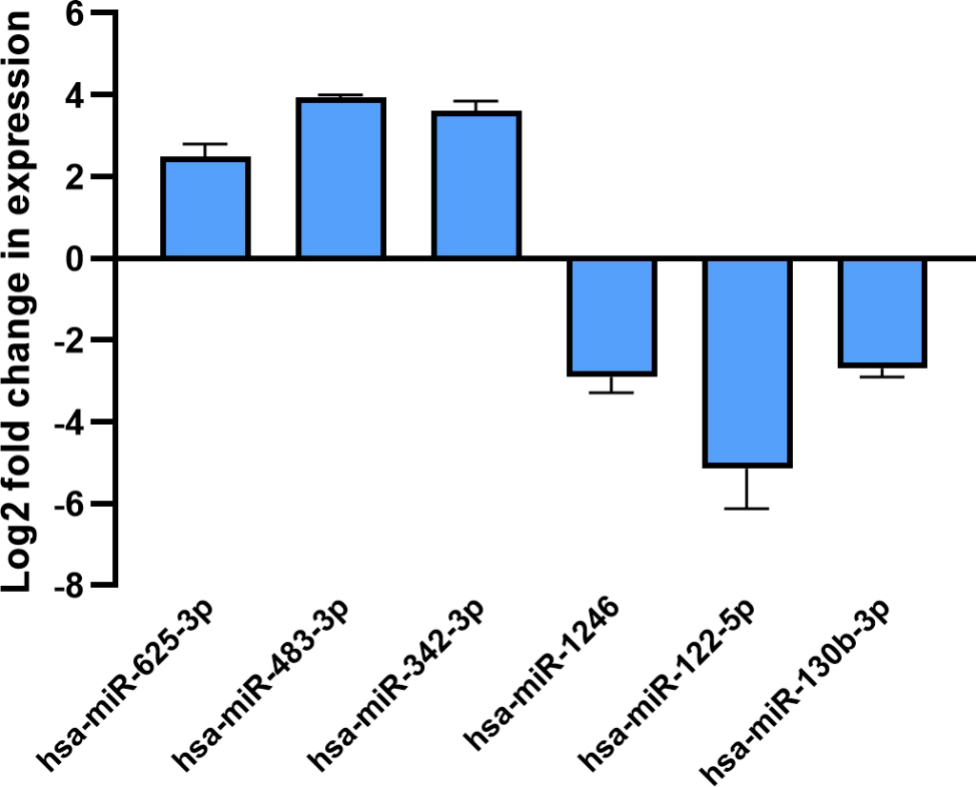
Validation of differentially expressed miRNAs in follicle fluid-derived exosomes

To evaluate whether the expression profile of exosomal miRNA in the sample was consistent with deep sequencing data, six DEmiRs (hsa-miR-625-3p, hsa-miR-483-3p, hsa-miR-342-3p, hsa-miR-1246, hsa-miR-122-5p, and hsa-miR-130b-3p) were validated using qRT-PCR. The results showed that the differential miRNA relative expression levels were generally consistent with the sequencing results (Fig. 6).

### Protein-protein interaction (PPI) network construction and Module analysis

**Fig. 7:**
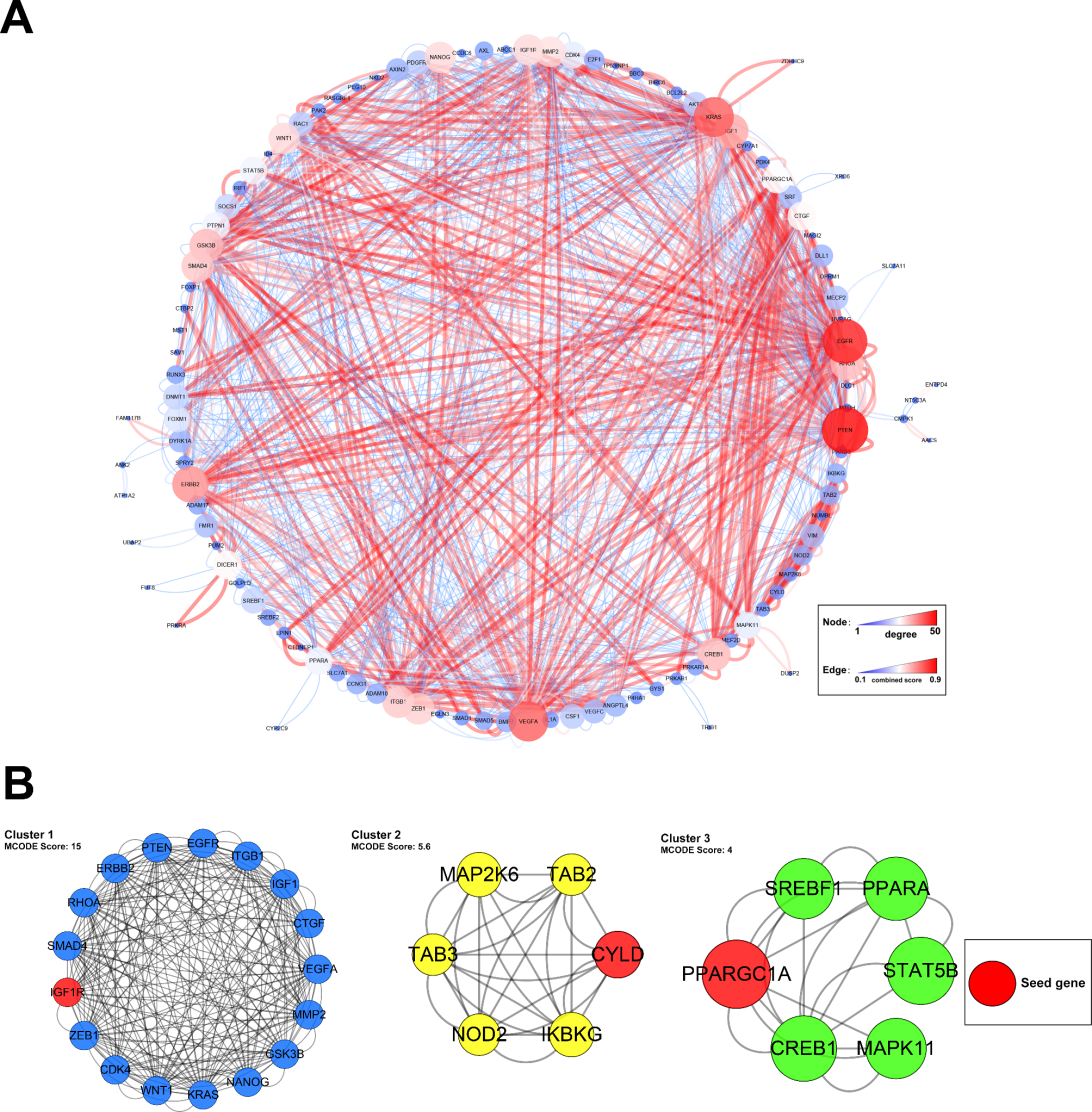
**(A)** The protein-protein interaction (PPI) analysis of the putative target genes of DEmiRs. The degree was used for describing the importance of protein nodes in the network, and the combined score of edge stands for the strength of interaction between two genes. **(B)** The significant three modules were identified from the PPI by using MCODE method

Data analysis was performed based on the STRING database. Out of 1142 protein pairs and 126 nodes from DEmiR target genes, a combined score > 0.4 was revealed. Several genes with the greatest degree values were discovered in the PPI network created using Cytoscape, including PTEN (degree = 50), EGFR (degree = 50), and KRAS (degree = 50) (Fig. 7A). Performing module analysis using the MCODE method, there were three module clusters in the PPI network. It was found six hub genes in Cluster 1 had a MCODE score > 6.0, including IGFR, VEGFA, KRAS, ERBB2, RHOA and PTEN (MCODE score = 11.472) (Fig. 7B).

## Discussion

Researchers have made substantial progress for DOR in understanding the biological mechanisms and pathogenesis agents. Updated work also demonstrates the importance of miRNAs in the pathophysiology of DOR. Numerous previous research studies [ 22, 23, 24 ] investigated the expression of specific miRNAs in cumulus cells or granulosa cells from patients with DOR. Furthermore, exosome information transmission was thought to be a crucial approach to cell-to-cell communication in ovarian follicle development [19]. However, little is known about exosomal miRNAs associated with ovarian follicle development and the pathogenesis of other ovarian diseases, especially DOR.

In this study, a comparison of exosomal miRNA expression profiles between Normal and DOR follicular fluid (FF) samples was initially explored using NGS sequencing. 19 up-regulated and 33 down-regulated exosomal DEmiRs were identified in DOR group. Further investigate the potential functions of exosomal miRNAs through GO, KEGG pathway, and DO annotation. GO analysis findings indicated that DEmiRs have significance to a range of biological processes, mainly including *cell migration involved in sprouting angiogenesis* (GO0002042), *cytosolic small ribosomal subunit* (GO0022627), *cell maturation* (GO0048469), and so on. It is commonly regarded that angiogenesis is crucial to folliculogenesis, ovulation, and luteinization. Qu Q et al. [25] discovered that hucMSCs-derived exosomal miR-126-3p may increase angiogenesis and inhibit ovarian granulosa cells (OGCs) apoptosis in a rat POI model. Recent research employing in silico analysis identified that a crucial dysregulated angiogenesis-related miRNA-mRNA network, which is involved in impaired follicular angiogenesis in PCOS [26]. The transmission of cell-secreted vesicles containing miRNAs between germ cells and their surrounding somatic cells, is indispensable for germ cell maturation [27]. Otherwise, numerous researchers have also suggested that exosomal miRNA may be a possible regulator of TGFB/BMP, WNT, MAPK, ErbB, and the ubiquitin-mediated signaling pathway for modulating mammalian follicle and oocyte maturation [18–20, 28]. It implies that these exosomal DEmiRs may be crucial in the pathological process of ovarian dysfunction in patients with DOR by regulating angiogenesis and cell maturation in the ovary. By analyzing 52 DEmiRs using the KEGG database, 104 pathways, such as *Fc gamma R-mediated phagocytosis*, *Inflammatory bowel disease IBD, mTOR signaling pathway*, were shown to be considerably enriched. Yefimova MG et al. [29] suggested an unconventional autophagy-assisted phagocytosis way to eliminate apoptotic oocytes by granulosa cells, which may be associated with ovarian dysfunctions caused by an imbalance in the content of germ cells in the ovaries. Several studies have determined that inhibition of the PI3K/AKT/mTOR pathway, which leads to the suppression of primordial follicle activity and a reduction in early-growing follicle apoptosis, may contributed to the protection of ovarian reserve and fertility potential [30, 31]. Results of disease ontology analysis show several significant highly expression up-regulated DEmiRs (hsa-miR-483-3p, hsa-miR-342-3p, hsa-miR-625-3p, and hsa-miR-532-3p) in DOR sample were closely related to ovarian neoplasms or carcinoma. In particular, hsa-miR-483-3p, which has the highest expression of all, has been verified to be related to cell proliferation and apoptosis in various cancers and other diseases [32, 33]. Based on reports and our experimental results, there is a reason to suspect that these DEmiRs play an essential role in the pathological process of ovarian reserve decreased.

To go further in the molecular mechanism of exosomal miRNAs, the top ten expression levels of DEmiRs in DOR sample (hsa-miR-1246, hsa-miR-483-3p, hsa-miR-122-5p, hsa-miR-130b-3p, hsa-miR-342-3p, hsa-miR-625-3p, hsa-miR-675-3p, hsa-miR-134-5p, hsa-miR-532-3p, and hsa-miR-1273f) were selected for searching potential target genes via miRanda, TargetScan and miRbase databases. According to miRNA-mRNA interaction data from online prediction tools, eight experimentally supported DEmiRs (hsa-miR-1246, hsa-miR-483-3p, hsa-miR-122-5p, hsa-miR-130b-3p, hsa-miR-342-3p, hsa-miR-625-3p, hsa-miR-675-3p, and hsa-miR-134-5p) and 126 target genes were discovered in the miRNA-mRNA network, and miR-342-3p and miR-122-5p were identified as having higher edge counts than other up- or down-regulated DEmiRs, respectively. It infers that exosomal miR-342-3p and miR-122-5p could be the key regulators of ovarian function and development. For instance, miR-122-5p, which targets BCL-9 to induce granulosa cell apoptosis, was overexpressed in premature ovarian insufficiency (POI) ovarian-derived exosomes [34]. In our results, the expression of miR-122-5p was greater in normal FF exosomes than in DOR. This phenomenon suggests that there is a different exosomal miRNAs-relate regulatory pathway between POI and DOR. Wang C et al. [35] determined that miR-342-3p could inhibit migration and invasion of ovarian carcinoma cells by downregulating FOXQ1. Another study has indicated that miR-342-3p regulates mammalian oocyte meiotic maturation by targeting DNA methyltransferase 1 [36]. Additionally, overexpression of miR-342-3p might be ascribed to the procession of ovulation [37]. To sum up, our findings prompt that there exists a unique exosomal miRNA expression profile for DOR, which is an important distinction from other types of ovarian dysfunction, and exosomal miRNA may also have a role in a variety of biological processes related to female fertility potential.

It is generally recognized that the function of miRNA depends on their downstream target genes. To further exploit the molecular mechanism of these miRNAs, PPI network analysis was performed to discover the hub genes in the downstream regulatory network. A total of three clusters were identified by using the MCODE method, but only cluster 1 had a score > 6.0 (MCODE score = 15). In cluster 1, the seed gene IGFR and the other five genes have the same score (MCODE score = 11.472) (VEGFA, KRAS, ERBB2, RHOA, and PTEN). Insulin-like growth factor 1 receptor (IGFR) has already been established to participate in the regulation of folliculogenesis and ovarian diseases [38, 39]. Recently, exosome-mediated transfer of non-coding RNA has been verified to be involved in angiogenesis [40], cell proliferation and fibrosis [41], and carcinoma progression [42] by regulating IGF1R. Vascular endothelial growth factor-A (VEGFA) is a pro-angiogenic cytokine and a cell survival factor, which is well established as the primary factor driving an expansion of the tumour vascular bed. Meanwhile, VEGFA also takes part in the process of ovarian cancer and follicle development by modulating the progression of angiogenesis, autophagy, and apoptosis [43, 44]. A functional role of phosphatase and tensin homolog deleted on chromosome 10 (PTEN) in the regulation of ovarian function has been extensively studied, and it is well known as an important modifier of the biological functions of ovarian granulosa cells (GCs) [45]. The latest research suggested that miR-18b-5p, which was produced by follicular fluid-derived exosomes, reduces PTEN expression and promotes the activation of the PI3K/Akt/mTOR signaling pathway to improve polycystic ovary syndrome (PCOS) development [46], and Eman Thabet et al. [47] found that extracellular vesicles miRNA-21 promoted ovarian restoration by modulating PTEN and caspase 3 apoptotic pathways. Otherwise, KRAS, ERBB2, and RHOA are all in connection with ovarian cancer [48–50], but little is known about the mechanisms involved in other ovarian functional regulation of these genes, and there are also only a few exosomal miRNA-related pieces of research. To summarize, we found some important DEmiRs and their downstream target genes, which may be potential biomarkers or key regulators leading to a decrease in ovarian reserve.

We had made a lot of efforts to explore the differentially expressed follicular fluid-derived exosomal miRNAs in patients with diminished ovarian reserve; however, there are some limitations to our study: (1) It is essential to increase clinical samples to further observe and analyze whether differentially expressed miRNA may be employed as a diagnostic marker in the assessment of female patients with ovarian dysfunction. (2) We did not identify the specific origin of exosomes, which made us less understand how follicular fluid-derived exosomes affect the biological function of different cells in the ovarian follicle. (3) Although we detected certain critical exosomal miRNAs, such as miR-483-3p, but still require verification on the mechanism of the exosomal miRNA transferring pathway in the regulation of ovarian function.

## Conclusions

In conclusion, we have described the specific expression profile of follicular fluid-derived exosomal miRNAs in patients with DOR and identified the differentially expressed miRNAs and their target genes that could be associated with ovarian dysfunction and follicle development. Further studies will try to determine whether any specific miRNA or combination of miRNAs has an influence on the pathogenesis of DOR or whether they may represent potential biomarkers of the disease or treatment response. In addition, we would like to investigate the biological function of exosomal genetic material or proteins in the follicle microenvironment to further gain a deep insight into the molecular mechanism of infertility-related ovarian diseases.

### Electronic supplementary material

Below is the link to the electronic supplementary material.

Supplementary Material 1

Supplementary Material 2

Supplementary Material 3

## Data Availability

The datasets generated and/or analysed during the current study are available in the NCBI SRA repository, (https://www.ncbi.nlm.nih.gov/bioproject/PRJNA906971/) (Accession: PRJNA906971).

## Abbreviations

DOR: Diminished ovarian reserve
FF: Follicular fluid
NGS: Next-generation sequencing
DEmiR: Differentially expressed microRNA
POI: Premature ovarian insufficiency
