# Supplementary figures and images for: Specific expression profile of follicular fluid-derived exosomal microRNAs in patients with diminished ovarian reserve
Source: BMC Med Genomics. 2023 Nov 30;16:308. doi: 10.1186/s12920-023-01756-9 (PMC10688486; doi:10.1186/s12920-023-01756-9)

**Fig. S1**


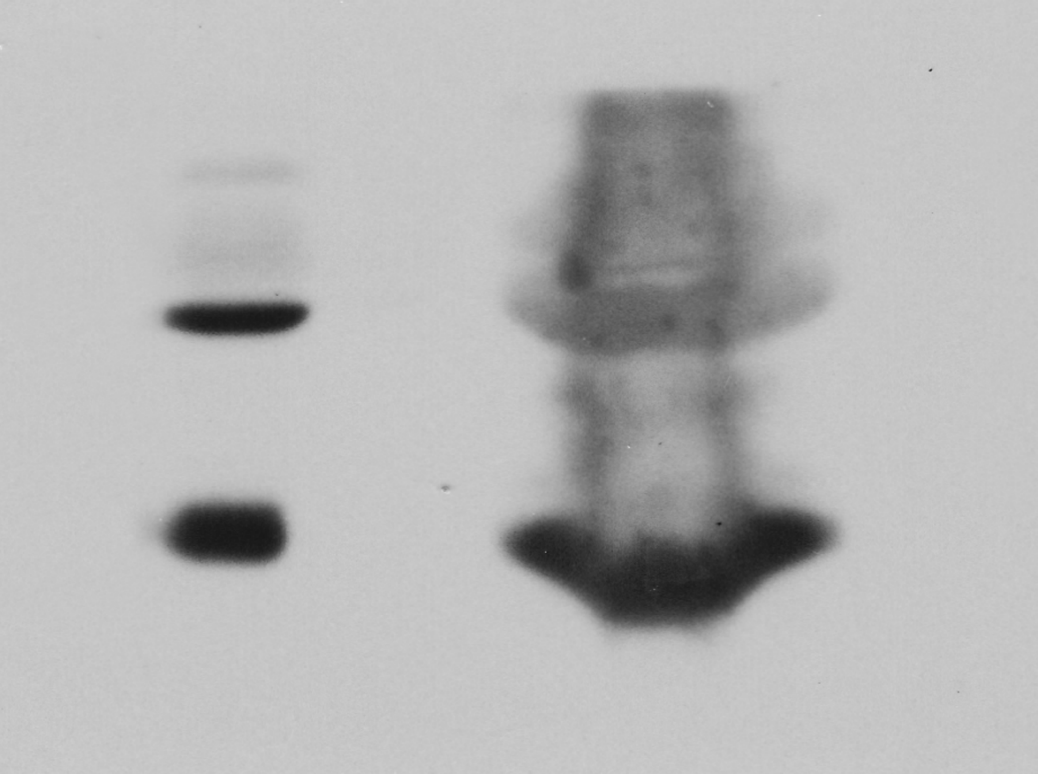

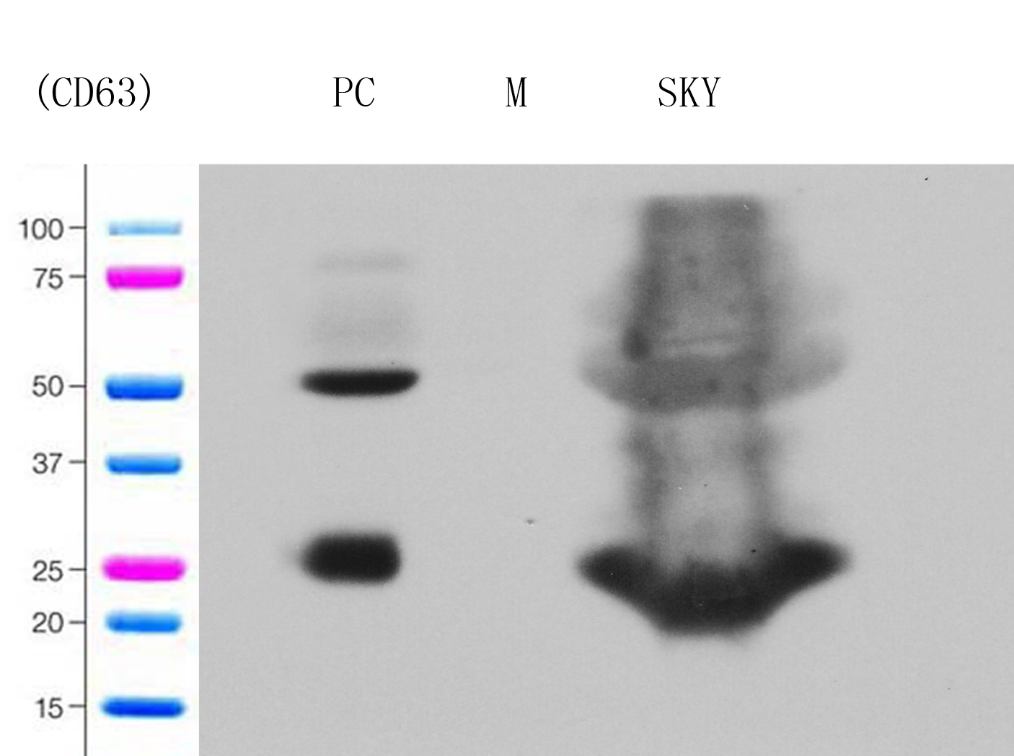


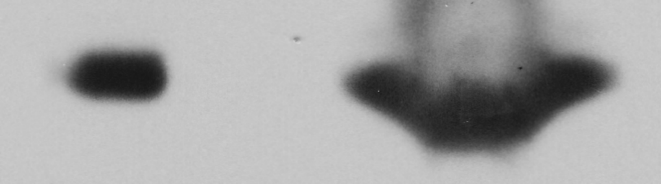


**Fig. S2**


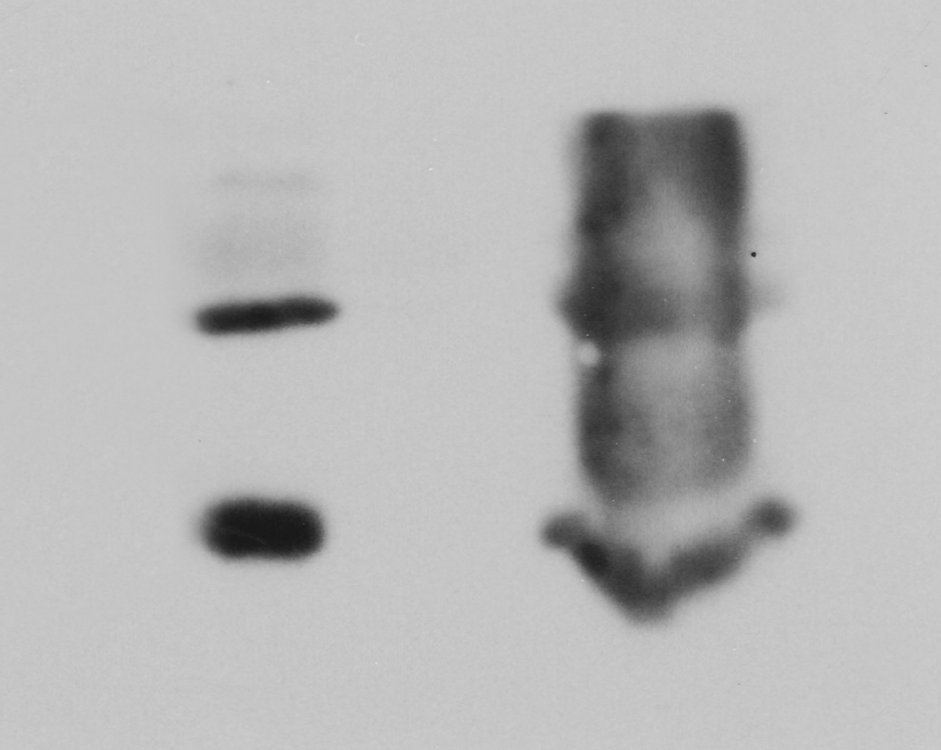

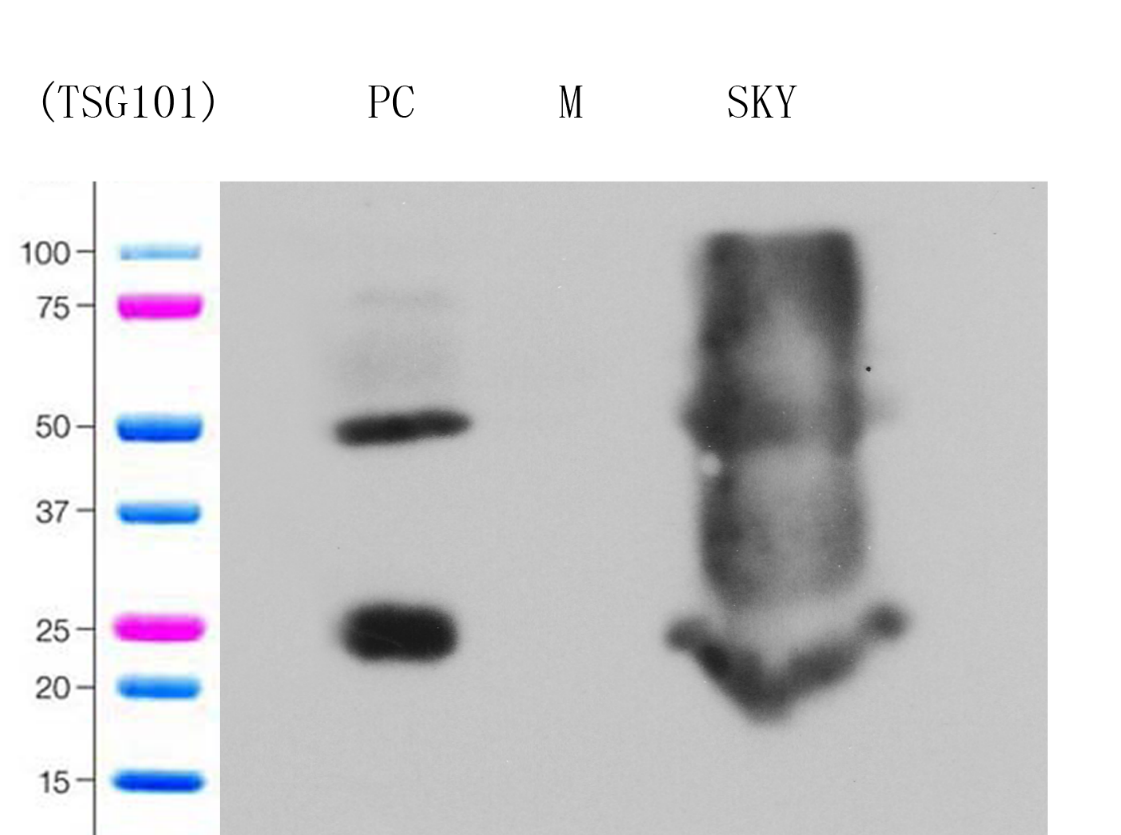


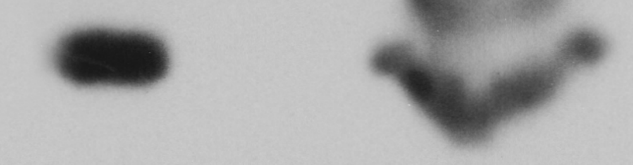


**Fig. S3**


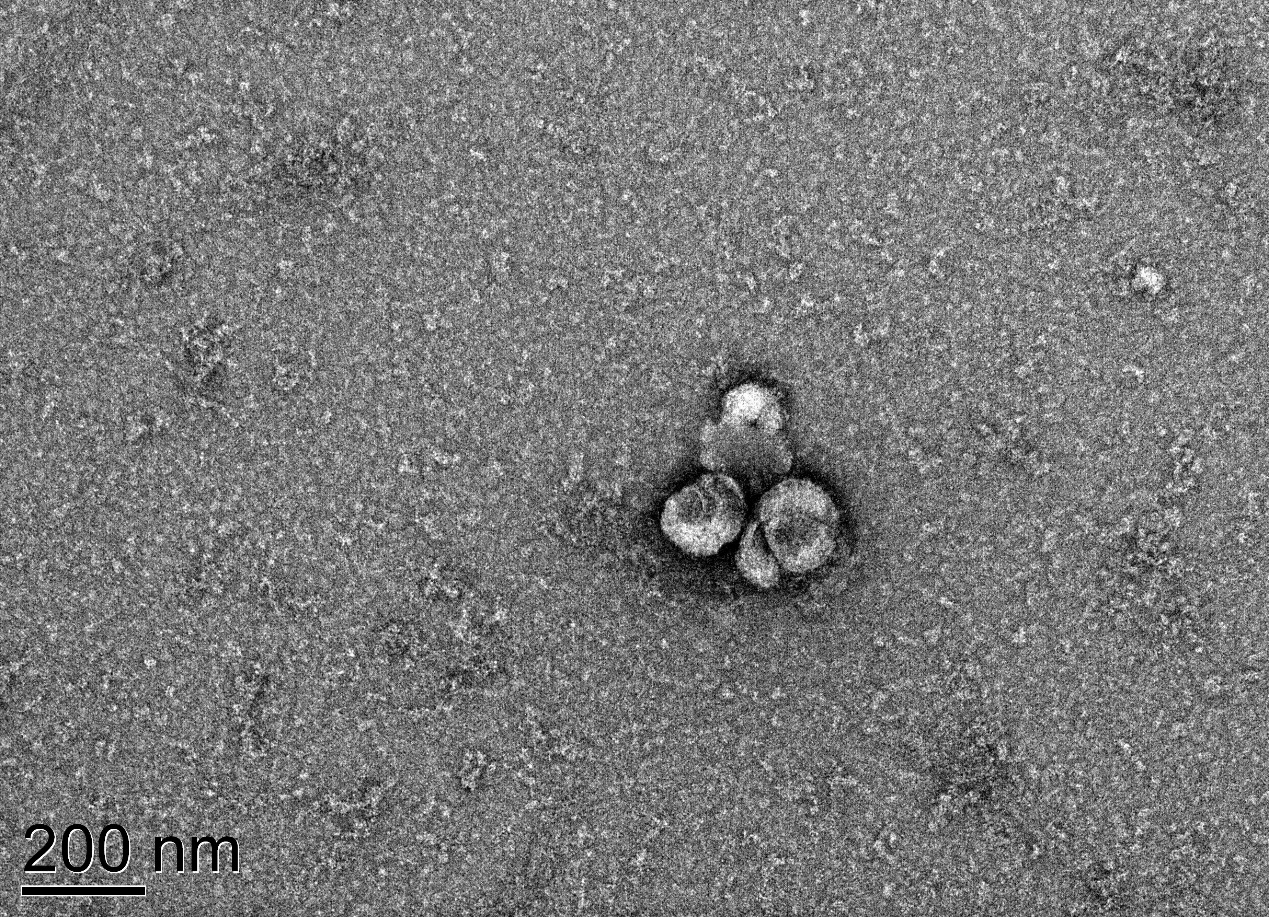


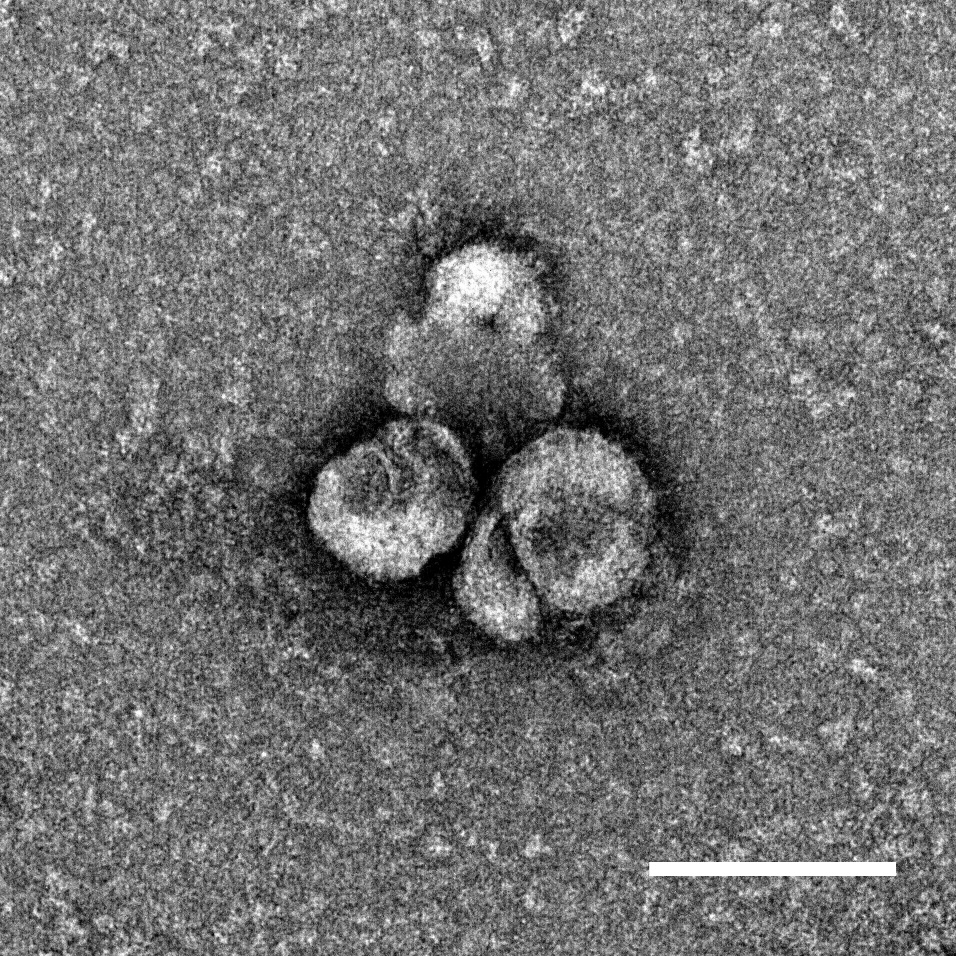

Supplement: Supplementary file 2 — Supplementary Material 2 [file 12920_2023_1756_MOESM2_ESM.docx]
