# Supplementary material for: Specific expression profile of follicular fluid-derived exosomal microRNAs in patients with diminished ovarian reserve
Source: BMC Med Genomics. 2023 Nov 30;16:308. doi: 10.1186/s12920-023-01756-9 (PMC10688486; doi:10.1186/s12920-023-01756-9)
Supplement: Supplementary file 3 — Supplementary Material 3 [file 12920_2023_1756_MOESM3_ESM.docx]

**Additional files legends**

**Additional file 1. Supplementary tables S1–S5.**

**Table S1.** The raw data for the identification of differentially expressed microRNA by NGS analysis.

**Table S2.** Gene onthology analysis result of the differentially expressed microRNA

**Table S3.** KEGG pathway analysis result of the differentially expressed microRNA

**Table S4.** Disease onthology analysis result of the differentially expressed microRNA

**Table S5.** qRT-PCR primers for follicular fluid-derived exosomal miRNAs.

Additional file 1 is submitted as xlsx file, you can use Microsoft office Excel or other suitable software to open it.

**Additional file 2. Supplementary figures S1, S2 and S3.**

**Fig S1.** West blotting original image of CD63

**Fig S2.** West blotting original image of TSG101

Both of CD63 and TSG 101 blotting gels contain 3 lanes, lane 1: positive sample, lane 2: Marker, lane 3: exosome protein sample.

**Fig S3.** Transmission electron microscopy original image of exosome
